# Making healthy homes? A pilot study of the return on investment from an external wall insulation intervention

**DOI:** 10.1186/s13104-017-3067-x

**Published:** 2017-12-13

**Authors:** Heather Brown, Gulnar Fattakhova, Clare Bambra, Paul Taylor

**Affiliations:** 1 Newcastle University, Institute of Health and Society, Newcastle upon Tyne, NE2 4AA UK; 2 Principal Environmental Officer, Economic Growth & Development, Stockton on Tees Borough Council, Municipal Buildings, Church Road, Stockton, TS18 LTD UK

**Keywords:** External wall insulation, Fuel poverty, Health related quality of life, Return on investment analysis, UK, Pilot study

## Abstract

**Objectives:**

External Wall Insulation (EWI) insulates and protect homes against damp. The Energy Company Obligation (ECO) scheme incentivised large energy providers in the UK delivering energy efficiency measures such as EWI to fuel impoverished households. Return on Investment (ROI) analysis is utilised to determine if EWI is a cost-effective procedure in terms of improving health related quality of life (HRQOL) measured using the EQ-5D-3L™, reducing health care expenditure, and fuel costs. Data comes from Stockton-On-Tees council, health care costs data, and information collected from households in the most socially deprived areas in Stockton-on-Tees.

**Results:**

The total cost of installation across all 2252 that received EWI was £10,222,954 in 2016 GBP. Annual total benefits were extrapolated across all 3265 households that received EWI. Total benefits were differences between the control and treatment groups in fuel costs, health care costs, and HRQOL multiplied by the National Institute for Health and Care Excellence Quality Adjusted Life Year threshold (£20,000). Total benefits for all households that received EWI were £1,519,045. The ROI of EWI is − 41%. 7.9 years are needed to recoup the costs of the initial investment.

**Electronic supplementary material:**

The online version of this article (10.1186/s13104-017-3067-x) contains supplementary material, which is available to authorized users.

## Introduction

Poor housing has a detrimental effect on health costing the National Health Service (NHS) at least £600 million per year [1]. Housing related hazards that increase the risk of illness stem from damp, mould, and excess cold [2]. External Wall Insulation (EWI) is a thermally insulated exterior wall cladding procedure that can be used to insulate homes and protect against damp. EWI is the most cost efficient way to insulate solid wall homes. More than 45% of all fuel poor households live in solid wall properties and approximately 20% of all households living in solid wall housing are living in fuel poverty. [3] Wider benefits of EWI in terms of improved respiratory and cardiovascular health and subsequently reduced costs of treating these conditions, area level regeneration, aesthetic improvements, and social capital have been identified. [3] There is no evidence investigating whether EWI may provide wider health benefits related to mental and physical health. In 2012, The Energy Company Obligation (ECO) scheme was designed to incentivise large energy providers in the UK to fund and install energy efficiency measures such as EWI to the most fuel impoverished households [4]. Understanding how the installation of EWI through the ECO scheme will benefit the most deprived households is important for reducing inequalities and improving health.

In this study we report on a pilot study to evaluate the ECO scheme in Stockton-on-Tees an area level installation of EWI of 3265 homes in eight of the most socially deprived lower super output areas (LSOAs)^1^ with high incidence of fuel poor and fuel poverty households in Stockton-On-Tees, UK. We perform a Social Return on Investment Analysis (ROI) to evaluate if EWI is cost-effective in terms of the return to health related quality of life (HRQOL) measured using the EQ-5D-3L™^2^ [5], health care expenditure, and fuel spending.

## Main text

### Methods

The primary source of data for this project came from a cross-sectional postal survey containing questions on HRQOL on the day of the survey, measured using a standardised EQ-5D-3L™ tool [5] healthcare usage, fuel spending, and demographic information such as household income, age, and gender. Additional file 1 is the questionnaire sent to households. Sample sizes were determined by the number of properties that had received EWI in the most deprived areas of Stockton and the control group were houses that would have been eligible for EWI if the Eco Scheme had continued and had similar socioeconomic status, a similar housing stock, and were located within similar lower layer super output area (LSOA). Questionnaires were posted to a total of 3256 household consisting of 1149 households that received EWI in 2012 (early cladders) and 1103 households that received EWI in 2014–2015 (late cladders), and a control group of 1004 households that had not received EWI but had similar socioeconomic and housing characteristics to the intervention groups. The response rate to the questionnaire was approximately 7% (n = 232). From intervention group 1 (early cladders) n = 91, n = 78 respondents from intervention group 2 (late cladders) and n = 63 from the control group.

Additional data on the costs of installing EWI was provided by Stockton-On-Tees Borough Council, UK. To quantify any observed differences in health expenditure between those who had received EWI and the control group, Information on cost of health care usage was taken from the Unit Costs of Health and Social Care 2015 [6] and National Schedule of Reference Costs 2014–2015 (Additional file 1). [7] Costs for prescriptions was taken from the British National Formulary 2016. [8] All costs were presented in 2016 Great British Pounds (GBP).

Data was analysed using the statistical software package, STATA v.14. [9] To conduct the ROI, we needed to estimate the total costs to compare with the total benefits of EWI to recipients. Total costs were estimated as the mean cost of EWI per household multiplied by the number of households which received EWI. There were no maintenance costs of EWI in the first 4 years after installation. The company that installed EWI provided a 25 year warranty of works and materials and therefore there is no cost to the household of the insulation during this period. To estimate total benefits, firstly we estimated mean differences in fuel spending, HRQOL measured as mean difference in total EQ-5D-3L score, and mean difference in health care expenditure between the control group and the early cladder group. These models were estimated using Ordinary Least Squares (OLS) and controlled for age, gender, and household income which may impact on our benefit outcomes of interest. Next, to provide meaningful values for the ROI, the adjusted mean differences were further manipulated. Mean adjusted fuel expenditure was multiplying by 12 (to estimate costs over a whole year) and then multiplying again by the total number of households. Mean adjusted HRQOL was multiplied by £20,000—the maximum value that the National Institute of Health and Clinical Excellence (NICE) (which makes recommendations on services and treatments which should be funded by the NHS) will pay for a quality adjusted life year [10] and multiplied by the total number of households. Health care costs were estimated as adjusted difference between early cladders and control group multiplied by number of individuals requiring health care treatment multiplied by cost of treatment/medicine. These amounts were summed to provide annual total benefits and benefits over a 4 year period (the time that had elapsed since early cladders received EWI). Finally, we estimated the ROI model over the 4 year period since the early cladders received EWI which was calculated by Eq. (1):

1 $$ROI = \frac{(Total\,Benefits - Total\,Costs)}{Total\,Costs}$$

### Results

**Fig. 1:**
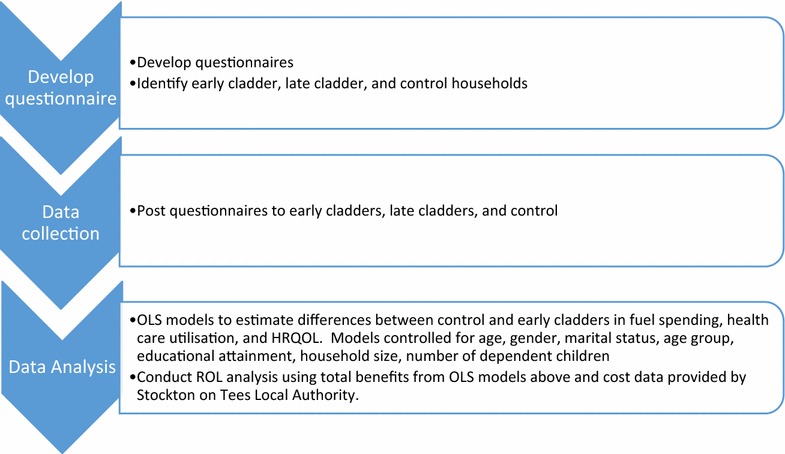
Research plan

The Research plan is shown in Fig. 1. Table 1 shows the total costs and benefits of EWI which were used to estimate the ROI. In column 1, we can see the costs of installing EWI. The average cost of delivering the intervention per household is £4539.50 in 2016 GBP. The total cost of delivering the intervention to the 2252 households which received EWI is £10,222,954 measured in 2016 GBP. Annual total benefits which were comprised of adjusted differences in fuel expenditure, HRQOL, and health expenditure between the early cladders and control group were £1,519,045 measured in 2016 GBP. This amount is extrapolated across all households and includes reductions in fuel expenditure, health care costs, and improvements in health related quality of life multiplied by the quality adjusted life year (QALY) threshold which is £20,000 for the UK compared to the control group. Benefits over the 4 year period since the first set of household received EWI were calculated to be £6,076,180 in 2016 GBP. The ROI of EWI in relation to HRQOL, health care and fuel expenditure is − 41%. This suggests it will take 7.9 years to recoup the costs of the initial investment (Table 1).

**Table 1:** Return on investment

Costs (£)	Benefits (£)		ROI
Project implementation = £4539.50 (cost of installation per house) * 2252 (# of households receiving EWI) = £10,222,954	Adjusted monthly difference in fuel expenditure (early cladders-control) 40	Annual adjusted difference between treatment and control group * total households £40 * 12 * 3256 = *£1,562,880*	
Maintenance = £0 (over first 4 years of installation)	Adjusted difference in EQ-5D-3L score (early cladders-control) 0.01	Adjusted Difference in EQ-5D-3L score * NICE QALY threshold * total households 0.01 * £20,000 * 3256 = *£651,200*	
	Adjusted difference in usage * cost of health care (early cladders-control): outpatient appointments/hospital visits (£4,185,665–£3,111,284) Medical procedures: (£887,609–£1,159,201) Medication: (£60,254–£4,438,493)	Sum of adjusted difference in health care costs between early cladders and control: *−* *£695,035*	
*Total costs* *=* *£10,222,954*	*Total benefits* *=* *£1,519,045/year or* *£6,076,180 for 4* *years*		ROI (%) = (benefits-costs)/costs = *−* *41%*

Total amounts are in italics

### Discussion

We employed a ROI to provide some preliminary evidence if EWI may be a cost-effective measure to improve HRQOL, reduce health expenditure, as well as reducing fuel poverty measured by fuel expenditure in socioeconomically disadvantaged areas with poor housing stock. Living in a consistently under-heated home poses significant health risks through increased incidence of damp and mould [11]. The cost reductions of EWI on cardiovascular and respiratory illness, conditions typically associated with living in cold and damp conditions [12], has been estimated at £183 million per annum [3]. If there are wider health benefits in terms of improving HRQOL and reductions in health expenditure, as our results suggest, this would imply that the health benefits to the NHS may be even greater. Our finding of a reduction in monthly fuel expenditure of £40 is similar to larger evaluations of the benefits of EWI [3]. This boost in household income will be important for socially deprived households. Our results from a small sample from Stockton-On-Tees provides support for future research investigating how EWI may improve health and provide wider benefits than those which have traditionally been focused upon.

### Conclusion

Tackling fuel poverty and inadequate housing requires a multidisciplinary approach. This research drew upon expertise in public health, geography, health economics, and local government. Accessing data from households with similar characteristics that have received EWI to a control group which has not received EWI but would have been eligible for EWI if the Eco Scheme had continued has allowed us to provide preliminary evidence if EWI may be cost-effective in relation to health and fuel poverty.

The Scheme in Stockton-on-Tees funded via ECO to provide EWI to households in the most deprived LSOAs has been found to reduce fuel expenditure and provided preliminary support for improving HRQOL. A long term outlook is required for making informed decisions regarding all the potential public health benefits of EWI. This will be used to inform future work in this area.

## Limitations

The response rate for our questionnaire was low (7%). Financial resources constrained us to a postal survey which may partially explain the low response rate. Evidence from the UK suggests that low response rates to postal questionnaires in socially deprived areas such as Stockton-On-Tees, stems from disengagement, low literacy rates, and poor contact information [13]. Research shows that civic participation does not differ by socioeconomic status or ethnicity in the UK [13]. The low participation rate may have biased our findings. Nevertheless, our results are important for informing the direction of future research in the area to collaborate these findings. For a further evaluation, we plan to utilise alternative methods such as door to door or telephone questionnaires to improve the response rate for this hard to reach group. In addition, the study is confined to one city in the North East of England; thus, it is possible that climate may affect the generalisability of these findings to other areas.

## Additional file

**Additional file 1.** Health care usage.

## Footnotes

1 The smallest geographical area for which census estimates are provided.

2 EQ-5D-3L is an assessment tool for measuring HRQOL and is a paper based questionnaire asking respondents about five dimensions of their health: (1) mobility; (2) self-care; (3) usual activities; (4) pain/discomfort; and (5) anxiety/depression. Each dimension has three levels: (1) no problems; (2) some problems; and (3) extreme problems.
