# Supplementary material for: Making healthy homes? A pilot study of the return on investment from an external wall insulation intervention
Source: BMC Res Notes. 2017 Dec 13;10:736. doi: 10.1186/s13104-017-3067-x (PMC5729294; doi:10.1186/s13104-017-3067-x)
Supplement: Supplementary file 1 — Additional file 1. Health care usage. [file 13104_2017_3067_MOESM1_ESM.docx]

**Section 1: Health Care Usage**

Please complete this section on your health care usage over the past six months.

Please tick the appropriate boxes and answer the questions where required.

| THIS SET OF QUESTIONS IS ABOUT ANY APPOINTMENTS YOU MAY HAVE HAD WITH A GENERAL PRACTICE IN THE PAST 6 MONTHS |
| --- |

| **1.** Have you consulted a GP for any reason during the last 6 months? |  |
| --- | --- |

| Yes **If Yes, go to Q1a**  No **If No, go to Q2** |  | | |
| --- | --- | --- | --- |
|  | | |  |
| **1a.** How many appointments did you attend with a GP at their practice in the last six months? | |  |  |

| **1b.** How many times did a GP visit you at home in the last six months? |
| --- |

| **1c.** How many times did you have a telephone conversation with a GP in the last six months? |
| --- |

| **THIS SET OF QUESTIONS IS ABOUT ANY APPOINTMENTS YOU MAY HAVE HAD WITH other health care workers IN THE PAST 6 MONTHS** |
| --- |

| **2.** During the last **6** months have you had an appointment with: | | | | |
| --- | --- | --- | --- | --- |
|  |  |  |  |  |
| A Practice Nurse? | Yes |  | If Yes, how many appointments did you have? |  |
|  | No |  |  |  |
|  |  |  |  |  |
|  |  |  |  |  |
| Other? (*please specify*) | Yes |  |  |  |
|  | No |  |  |  |
|  |  |  |  |  |
|  | | | How many appointments did you have? |  |
|  |  |  |  |  |
|  | | | How many appointments did you have? |  |

| **THIS SET OF QUESTIONS IS ABOUT ANY MEDICATION OR MEDICAL PROCEDURES YOU MAY HAVE HAD IN THE PAST 6 MONTHS** |
| --- |

**3.** In the last **6** months, have you had any medical tests or procedures? (e.g. blood tests)

Yes **If Yes, go to Q3a**

No **If No, go to Q4**

**3a.** **If you have had any medical tests or procedures in the past 6 months, please provide details below:**

**Test Number of tests**

1. _______________________________________________

2. _______________________________________________

3. _______________________________________________

4. _______________________________________________

5. _______________________________________________

**4.** Are you taking any medication daily?

Yes **If Yes, go to 4a**

No **If No, go to 5**

**4a.** If Yes, please list the medications you are currently taking below:

**Medication**

1. _______________________________________________

2. _______________________________________________

3. _______________________________________________

4. _______________________________________________

5. _______________________________________________

| THIS SET OF QUESTIONS IS ABOUT ANY APPOINTMENTS YOU MAY HAVE HAD AT THE HOSPITAL IN THE PAST 6 MONTHS |
| --- |

**5.** In the last **6** months, have you had an admission to hospital as an inpatient?

Yes **If Yes, go to Q5a**

No **If No, go to Q6**

**5a.** If Yes, approximately how many nights in total did you spend in hospital in the last **6** months (if you were only admitted as a day case enter 0)?

Enter number of nights that you stayed in hospital

**6.** In the last **6** months, have you had any outpatient hospital clinic appointments?

Yes **If Yes, go to Q6a**

No **If No, go to section 2**

**6a.** If Yes, approximately how many hospital clinic appointments in total did you have in the last **6** months?

Enter number of times you attended hospital clinic appointments

**Section 2: Fuel Usage**

| THIS SET OF QUESTIONS IS ABOUT YOUR FUEL USAGE OVER THE PAST 1 MONTH |
| --- |

**1.** In the last month how much have you spent on fuel (gas and electricity) to heat and power the home?

**If possible please indicate gas and electricity costs separately**

**Put cost here:**

1. Amount spent on gas:

£

2. Amount spent on electricity:

£

3. Total amount spent on fuel to heat

£

and power the home:

**1b.** In the last month have you been able to keep the house heated to a comfortable temperature?

Yes **If Yes, go to Section 3**

No **If No, go to Q1c**

**1c.** If NO, how many days in the past month have you not been able to keep the house heated to a comfortable temperature?

**Please list number of days below:**

___________________________

**Section 3: General Health**

| THIS SET OF QUESTIONS IS ABOUT YOUR GENERAL HEALTH TODAY |
| --- |
| By placing a tick in one box in each group below, please indicate which statements best describe your own health state today. |

|  |  |
| --- | --- |
| Mobility |  |
| I have no problems in walking about | ❑ |
| I have some problems in walking about | ❑ |
| I am confined to bed | ❑ |
|  |  |
| Self-Care |  |
| I have no problems with self-care | ❑ |
| I have some problems washing or dressing myself | ❑ |
| I am unable to wash or dress myself | ❑ |
|  |  |
| Usual Activities *(e.g. work, study, housework, family or leisure activities)* |  |
| I have no problems with performing my usual activities | ❑ |
| I have some problems with performing my usual activities | ❑ |
| I am unable to perform my usual activities | ❑ |
|  |  |
| Pain / Discomfort |  |
| I have no pain or discomfort | ❑ |
| I have moderate pain or discomfort | ❑ |
| I have extreme pain or discomfort | ❑ |
|  |  |
| Anxiety / Depression |  |
| I am not anxious or depressed | ❑ |
| I am moderately anxious or depressed | ❑ |
| I am extremely anxious or depressed | ❑ |

|  |
| --- |
|  |
|  |
|  |
| To help people say how good or bad a health state is, we have drawn a scale (rather like a thermometer) on which the best state you can imagine is marked 100 and the worst state you can imagine is marked 0. |
|  |
| We would like you to indicate on this scale how good or bad your own health is today, in your opinion. Please do this by drawing a line from the box below to whichever point on the scale indicates how good or bad your health state is today. |

Best imaginable health state

Worst imaginable health state

9 0

8 0

7 0

6 0

5 0

4 0

3 0

2 0

1 0

100

0

Your own health state today

| OTHER INFORMATION |
| --- |

**Section 4: Demographic information**

1. Are you male or female?

Male

Female

1. Which age group do you belong to?

15-24

25-44

45-64

65-75

75+

1. What is your current marital status?

Married

Cohabiting

Separated

Divorced

Widowed

Never Married

1. What was your highest level of qualification when you finished your education?

Masters/PhD

1^st^ Degree

HND/HNC/Teaching

A level

GCSE /O-level

No Qualifications

Other

1. How many dependent children under the age of 16 are you responsible for?

**Number of children under the age of 16:**

___________________________

1. If applicable, what is the age of the **youngest** dependent child under the age of 16 that you are responsible for?

**Age of youngest dependent child:**

___________________________

1. Including dependent children, how many people are currently living at your household?

**Number of people in household:**

___________________________

1. What is your current employment status?

| Full Employment 🞎 | Part-time Employment 🞎 |
| --- | --- |
| Student 🞎 | Retired 🞎 |
| Housework 🞎 | Caring for someone 🞎 |
| Unemployed, not actively seeking work 🞎 | Unemployed, actively seeking work 🞎 |
| Other **Please provide details:__________________________________________** 🞎 | |

1. What is your weekly or monthly disposable household income (income remaining after taxes and national insurance contributions)?  Please provide a total for all household members.

**Total *weekly* disposable household income:**

**£**___________________________

**OR**

**Total *monthly* disposable household income:**

**£**__________________________
